# Level of Knowledge and Practice of Mothers on Minimum Dietary Diversity Practices and Associated Factors for 6–23-Month-Old Children in Adea Woreda, Oromia, Ethiopia

**DOI:** 10.1155/2017/7204562

**Published:** 2017-04-12

**Authors:** Andualem Agize, Dube Jara, Getiye Dejenu

**Affiliations:** ^1^College of Medicine and Health Science, Debre Markos University, Debre Markos, Ethiopia; ^2^Department of Public Health, College of Medicine and Health Science, Debre Markos University, Debre Markos, Ethiopia

## Abstract

*Background*. Globally, undernutrition is responsible for at least 35% of deaths in children less than 5 years of age and estimated 6% of under-five deaths can be prevented by ensuring optimal complementary feeding especially the dietary diversity and meal frequency. In Ethiopia, 5% of children were fed according to minimum standards with respect to food diversity.* Objective*. To assess the level of knowledge and practice of mothers on dietary diversity practices and associated factors for children 6–23 months in Adea woreda.* Methods*. Community-based cross-sectional study was conducted. A sample of 730 mothers who have children in the age group of 6–23 months were selected using systematic sampling. Logistic regression model was fitted in order to identify factors associated with knowledge and practice of dietary diversity practice.* Result*. Of the total 700, 357 (51%) were knowledgeable on dietary diversity but 112 (16%) practiced appropriate dietary diversity practice for their 6–23-month-old children. Husbands' education (AOR = 2.79, 95% CI = (1.55, 5.00)), mothers' age, and marital status were significantly associated with knowledge of mothers. Mothers' age, husbands' education, marital status, and knowledge of mothers were significantly associated with mothers' dietary diversity for 6–23-month-old children.* Conclusion*. This study showed that approximately half of the mothers have good knowledge on minimum dietary diversity for children 6–23 months old and very low proportion of children 6–23 months old received diversified meal according to Infant and Young Child Feeding indicators. It was identified that different factors are responsible for this discrepancy.

## 1. Introduction

The infant young child feeding model of World Health Organization (WHO) recommends introducing complementary food for infants starting at the sixth month. Breast milk has to be complemented by other foods to fill the nutrient demand of the child [1]. These complementary foods are needed to fill the calorie, protein, and micronutrient gap between the total nutritional need of the child and the amount provided by breast milk [2]. Appropriate complementary food has to be diversified and prepared from different food groups in a solid or semisolid form and needs to be initiated timely from the sixth month of the child's age by improving the quality of foods consumed as the child gets older [3].

Dietary diversity is defined as the number of the individual food items or groups consumed over a given period of time [3]. It can be measured at a household or an individual level through the use of questionnaire. More often, it is measured by counting the number of food groups [4]. Feeding practice during infancy is critical for growth, development, and health of the child and of importance for the early prevention of chronic degenerative disease [5]. Inappropriate complementary feeding increases the risk of undernutrition, illness, and mortality in infants and children [6].

Globally, undernutrition is responsible, directly or indirectly, for at least 35% of deaths in children less than five years of age. Undernutrition is also a major cause of disability preventing children who survive from reaching their full development potential. An estimated 32% or 186 million children below five years of age in developing countries are stunted and about 10% or 55 million are wasted [7].

An estimated 6% of under-five deaths can be prevented by ensuring optimal complementary feeding among which dietary diversity and meal frequency are the most important ones, significantly contributing to the realization of Millennium Development Goal 4 [8].

The lack of dietary diversity is a particularly severe problem among poor populations of developing countries because their diets are predominantly based on starchy staples and often include little or no animal products and few fresh fruits and vegetables. These plant-based diets tend to be low in a number of micronutrients, and the micronutrients they contain are often in a form that is not easily absorbed. Other aspects of dietary quality problems such as high intakes of fat, salt, and refined sugar in developed countries is becoming a concern for developing countries [9, 10].

Household level factors such as poor and middle household's socioeconomic status and lack of parental joint decision-making strategy on the treatment of the sick child and paternal education determine the nutritional status of the children. The lack of maternal access to health facilities and having a narrow birth interval and less dietary consumption from major food items were significantly associated with acute child undernutrition among the individual level factors [11].

Child dietary diversity practice was also associated with maternal dietary diversity practice. In the countries where maternal dietary diversity is high, the child dietary diversity practice is also high. In the countries where maternal dietary diversity is low, the child dietary diversity practice is also too low. In addition, the maternal power to control finance (earning money and buying food) was a key challenge to practice dietary diversity [12]. The case was not different in Ethiopia, particularly in Oromia region.

Therefore, the ultimate aim of this study will be to assess the level of knowledge and practice of mothers on dietary diversity practices and associated factors for 6–24-month-old children in Adea woreda, East Shoa Zone, Oromia, Ethiopia. The finding of this study will help policymakers and planners to set their target with feasible interventions in the study area as well as Ethiopia as a whole. It also reports on the existing gap for organizations and health workers working in the area, how to solve the problem, and how to design mechanisms for the next planning by providing information about children knowledge and practice of dietary diversity. The findings will also help zonal health department and regional health bureau to focus on identified problems in their future plan.

## 2. Methods and Materials

### 2.1. Study Setting and Period

The study was conducted from September 2014 to October 2014 in the Adea District, which is situated 45 km south of Addis Ababa and 50 KM from zonal capital Adama town. The Adea District comprises 23 kebeles with a total population of about 121,720. Over 90% of the district's population consists of subsistence farmers, depending heavily on rains for their agriculture. Maize, millet, cassava, and beans are the main crops cultivated in the area. There are five government-owned health centers and 23 health posts in charge of the whole population of the district. At* kebele* level, health posts are instituted with a health extension worker (HEW)* (source: Adea woreda Health Office, 2013)*.

### 2.2. Study Design and Population

Community-based cross-sectional study was conducted. The source population was mothers who have children in age group of 6–23 months who are living in Adea woreda. The study population was mothers who have children in the age range of 6–23 months who were selected to participate in the study. The mothers who were permanent resident of the study area were included in the study. Those mothers who are chronically ill were excluded from the study.

### 2.3. Sample Size and Sampling

The required sample size was determined using single population proportion formula. In this regard, 8.5% proportion of diversified dietary practice taken from previous studies conducted in northwest Ethiopia Enemay district, with a 3% margin of error and a 5% level of significance (two-sided), were assumed. Based on the above assumptions, considering a design effect of 2 since complex sampling (multistage) was used and adding 10% nonresponse rate, the total sample size was 730. Multistage sampling technique was used to selected the study subjects. Eight kebeles of the woreda were selected using simple random sampling from 23 kebeles. The calculated sample size was allocated to each selected kebele proportional to the size of the population and the study participants were selected using systematic random sampling through house number of each household of kebele.

### 2.4. Variables and Measurements

The dependent variable of the study was the knowledge and practice on minimum dietary diversity provision whereas the independent variables were sociodemographic variables (age, educational level, religion, marital status, ethnicity, family size, birth order, and season), socioeconomic characteristics (income, occupation, household economy control, livestock possession, exposure to information, etc.).

*Good Dietary Diversity.* Children 6–23 months of age who received foods from four or more food groups of the seven food groups [(1) grain, root, and tubers, (2) legumes and nuts, (3) dairy products, (4) flesh food, (5) egg, (6) vitamin A-rich fruit and vegetables, and (7) other fruit and vegetables] are used to have minimum dietary diversity [13].

*Knowledge.* Those who score above the average (mean) in knowledge assessing questions were categorized as knowledgeable and those who score below the average (mean) were categorized as not knowledgeable.

*Practice.* Those who fed their child with 4 or more food groups were categorized as practicing good dietary diversity (minimum acceptable diet) and below 4 groups were categorized as not practicing good dietary diversity.

*Kebele.* The smallest administrative unit of the country is called kebele.

### 2.5. Data Collection Methods

A structured questionnaire was developed based on previous studies and WHO recommendations to assess the dietary diversity by taking the local situation into consideration. The questionnaire was prepared in English and it was translated to Afan Oromo language (local language). Data were collected by trained data collectors and supervisors. The principal investigator organized a two-day training to introduce the objectives of the study and data collection process. The questionnaire was pretested out of the study area. The interview was administered in Afan Oromo language (local language). The data quality was assured by performing strong supervision by immediate supervisors. The supervisors also checked the completeness and consistency of questionnaires on daily basis. The principal investigator had also rechecked the completed questionnaires to maintain quality and has supervised five percent of the surveyed households to confirm whether the houses were interviewed in the intended way.

### 2.6. Data Processing and Analysis

The collected data were entered using EPI INFO statistical packages version 6.02. Then, they were exported to SPSS version 20 for analysis. The descriptive analysis was conducted to describe number and percentage distribution of variables. Binary logistic regression model was fitted to identify the association between outcome variable and explanatory variables. The crude and adjusted odds ratio together with its corresponding 95% confidence intervals was computed. A *P* value < 0.05 was considered to declare a result as statistically significant in this study.

### 2.7. Ethical Clearance

The ethical clearance was obtained from Debre Markos University College of Medicine and Health Sciences. A necessary permission was also obtained from Oromia regional health bureau and Adea woreda health office and local administrations. An informed verbal consent was obtained from the study participants and the privacy of the participants and confidentiality of the information were assured. Mothers with no and low awareness on dietary diversity for 6–23-month-old children were informed on the recommended practices and were referred to health facilities working nearby.

## 3. Results

### 3.1. Sociodemographic and Socioeconomic Characteristics of Respondents

Of a total of 730 sampled mothers, 700 were interviewed with an overall response rate of 95.9%. Four hundred thirty-seven (62.4%) of the respondents belonged to the age group of 25–34 years and five hundred fifteen (73.6%) of the respondents were Oromo and 176 (25.1%) were Amhara in ethnic group. The majority, 409 (58.4%), had children in the age group of 12 to 18 months. Four hundred twenty-four (60.6%) of the respondents attended their education to the primary level. Six hundred fifty-eight (94%) of the mothers were married and living with their husbands and 468 (66.6%) of the interviewed mothers' husbands attended their education to the primary level. Four hundred eighty-nine (69.6%) of the responding mothers were followers of orthodox Christian. Four hundred fifty-eight (65.4%) had family size of five and more members and 391 (55.9%) of the mothers had a monthly income of 501 to 1000 ETB. The household economy of six hundred forty-five (92%) mothers was controlled by husbands only (Table 1).

### 3.2. Knowledge of Mothers

The knowledge status of the mothers was assessed based on their self-responses. As a result, 357 (51%) respondents were knowledgeable on minimum dietary diversity practice. Six hundred seventy (95.7%) were knowledgeable on when to start complementary feeding. Four hundred one (57.3%) respondents breastfed their children for 12–23 months and 589 (84.1%) of the respondents were knowledgeable on why it is important to start giving their children food in addition to breast milk. In addition, 61 (8.7%) of respondents demonstrated the recommended thick porridge and 252 (36%) mentioned three and above food groups used to enrich a porridge. Regarding feeding methods, 572 (81.7%) participants reported feeding the child with a bottle as a recommended feeding option (Table 2).

### 3.3. Practice of Mothers in Provision of Diversified Diet

The majority, 663 (94.7%), of the respondents provided injera, bread, rice made of teff, oat, maize, barley, or wheat. Fourteen (2%) of the respondents provided juice or juice drinks and nine (1.35%) respondents provided organ meat like liver, kidney, and heart and sixty-eight (9.7%) respondents provided meat of ox, hen, sheep, or calf one day before the interview. Fourteen (2%) of respondents provided ripe mango or papaya and 23 (3.3%) of respondents provided pumpkin, carrot, squashed potato, or sweet potato that are yellow in color.

Generally, out of the seven hundred mothers interviewed, one hundred twelve (16%) responded that they have provided complementary food prepared of four and above food groups which is considered as good dietary practice (Table 3).

### 3.4. Factors associated with Knowledge of Mothers on Dietary Diversity Practice for 6–23-Month-Old Children

Maternal age, husbands' education, marital status, and source of health information had a statistically significant association with the outcome variables. Mothers whose age belongs to the age group of 35–44 years were 54% times less likely knowledgeable as compared with mothers whose age belongs to the age group of 15–24 years (AOR = 0.46, 95% CI = (0.26, 0.83)). Those mothers whose husbands' had attended their education to the secondary and above level were 2.79 times more knowledgeable on minimum dietary diversity for 6–23-month-old children as compared to those mothers whose husbands cannot read and write (AOR = 2.79, 95% CI = (1.55, 5.00)). Those mothers who were married and living with their husbands were 83% times less likely knowledgeable compared to single mothers (AOR = 0.17, 95% CI = (0.05, 0.56)). Mothers who responded that all food groups will be accessible in their household in winter were 1.46 times more knowledgeable as compared to mothers who will have access to all food groups in summer (AOR = 1.46, 95% CI = (1.01, 2.12)). Mothers whose monthly income ranges between 1001 and 3000 Ethiopian birr were 1.79 times more knowledgeable as compared with those whose monthly income ranges between 501 and 1000 birr (AOR = 1.79, 95% CI = (1.28, 2.51)).

Mothers who received health information from health centers were 53% times less knowledgeable as compared with mothers who received health information from health posts only (AOR = 0.47, 95% CI = (0.23, 0.93)). And mothers who received health information from health posts and media like radio and television were 2.25 times more knowledgeable as compared with mothers who received health information from health posts only (AOR = 2.25, 95% CI = (1.37, 3.96)) (Table 4).

### 3.5. Factors associated with Dietary Diversity Practice of Mothers of 6–23-Month-Old Children

Maternal age, husbands' education, marital status, seasonal availability of food, access to all food groups, source of health information, and knowledge of mother had a statistically significant association with outcome variable. Those mothers who belong to the age group of 25–34 years were 1.82 times more likely to practice good dietary diversity for their 6–23-month-old children as compared with those who belong to the age group of 15–24 years (AOR = 1.82, 95% CI = (1.05, 3.17)). Mothers who were married and living with their husbands were 88% times less likely to practice good dietary diversity as compared with single mothers (AOR = 0.22, 95% CI = (0.08, 0.59)). And those mothers who were divorced were 91% times less likely to practice good dietary diversity compared to single mothers (AOR = 0.09, 95% CI= (0.02, 0.53)). Mothers' dietary diversity practice was also affected by seasonal availability of food groups to diversify meal for their 6–23-month-old children. Mothers who can have access to all food groups in winter were 1.78 times more likely to practice good dietary diversity as compared with those mothers who can have access to all food groups in summer (AOR = 1.78, 95% CI = (1.01, 3.15)).

This study revealed that source of health information had also a significant effect on dietary diversity practice for 6–23-month-old children. Mothers who received health information from health posts and health development army (HDA) were 52% times less likely to practice good dietary diversity for their children as compared with those mothers who received health information from health posts only (AOR = 0.48, 95% CI = (0.29, 0.79)).

The knowledge of the mother was also computed as an explanatory variable for dietary diversity practice. Accordingly, mothers who were not knowledgeable on dietary diversity were 70% times less likely to practice good dietary diversity for 6–23-month-old children as compared with their counterparts (AOR = 0.30, 95% CI = (0.18, 0.48)) (Table 5).

## 4. Discussion

The feeding practice during infancy is critical for growth, development, and health of child and of importance for the early prevention of chronic degenerative disease [5]. Inappropriate complementary feeding increases the risk of undernutrition, illness, and mortality in infants and children [6]. Complementary foods are additional foods which are provided in addition to breast milk in order to fill the energy gap demanded by the children for proper growth and development. Therefore, the objective of this study is to assess the level of knowledge and practice of mothers on dietary diversity practices and associated factors for 6–23-month-old children in Adea woreda, East Shoa Zone, Oromia.

The finding of this study revealed that 51% of the respondents were knowledgeable on dietary diversity. About 95.7% of the respondents reported that a child has to start complementary food at the sixth month in addition to breast milk which was similar to the findings of the study conducted in Accra, Ghana [14]. A very low proportion of the mothers were knowledgeable on the consistency of complementary food for 6–23-month-old children, that they show pictures of thick porridge. About 36.1% of the respondents had mentioned three and above food groups which can be added to complementary food to enrich porridge and were categorized as knowledgeable. This finding is consistent with the report of the Food and Nutrition Technical Assistance [15].

Husbands' education status showed statistically significant association with mothers' knowledge on dietary diversity for 6–23-month-old children. The mothers whose husbands had had secondary and above education level were more knowledgeable on dietary diversity for 6–23-month-old children. This finding is consistent with the finding of the study conducted in Bangladesh [16].

Mothers who were married and living with their husbands were more knowledgeable as compared with single and divorced mothers. This can strengthen the above suggestion that husbands can discuss the children feeding with their wives. Household monthly income was significantly associated with mothers' knowledge on minimum dietary diversity. Mothers whose household income was high were more knowledgeable compared to mothers whose income was low and the practice was also higher and this finding is in line with that of the study done in India [17].

In this study, most of the respondents (99%) breastfed their children during the interview and 94.7% of respondents had provided grain-based food for their children, 48.9% have provided egg, and only 9.7% have provided flesh food like meat of ox, sheep, or hen. This finding was higher in the practice of dietary diversity when compared with the findings in Ethiopian Demographic and Health survey 2011 [18]. Though there is increment in the practice, still body building food group like flesh food consumption is very low. This might be due to knowledge gap on flesh food consumption or other sociodemographic characteristics of mothers.

This study revealed that 16% of the respondents practiced good dietary diversity (minimum dietary diversity) for 6–23-month-old children. This finding is higher as compared with the findings of studies on minimum dietary diversity practices for 6–23-month-old children conducted in Enemay woreda, North West Ethiopia, which was 8.5% (28) and with finding of Ethiopian Demographic and Health Survey 2011 which was 5% [18]. This might be due to the different Infant and Young Child Feeding interventions in the study woreda. The finding was almost similar to that of the study conducted in India in which the minimum dietary diversity practice was 15.2% [17]. This finding is very low compared to that of the study done in Bangladesh, Sri Lanka, Nepal, and Ghana in which the minimum dietary diversity practice for 6–23-month-old children was 41.9%, 71%, 34%, and 41.8%, respectively [16, 19–21]. This might be due to the economic status of the mothers in these countries.

Mothers' age was associated with child dietary diversity practice. Mothers whose age belongs to the age group of 25–34 years practiced good dietary diversity (minimum acceptable dietary diversity) than mothers that belong to any other age groups. This could be due to the increase of dietary diversity feeding practice as the age of the mother increases and the mothers gain experience in child feeding. Seasonal availability of food group was also found to be statistically significantly associated with dietary diversity practice for 6–23-month-old children. Dietary diversity practice was higher among respondents who can have access to all food groups in winter compared to in summer. This could be due to the increased access to all food groups by selling the household surplus production.

The source of health information was found to have statistically significant association with the minimum dietary diversity practice for 6–23-month-old children. The respondents who receive health information from health posts were practicing good dietary diversity (minimum dietary diversity) for their children compared to mothers who receive health information from health centers and messages from both health posts and health development army (HDA). This might be because, in the health posts (HP), health extension workers are providing more health education and give more time to discuss nutrition with mothers and the messages are clearly communicated with no language barrier. In addition, more preventive nutrition training sessions are offered for HEW and there is less turnover of the trained health extension workers (HEW) in HP than in health centers.

There was very high difference between the knowledge of the mothers on dietary diversity (51%) and minimum dietary diversity practice for 6–23-month-old children which was 16%. This finding is similar to that of the study conducted in Accra, Ghana [22]. This indicates and strengthens those different sociodemographic factors like mothers' age, husbands' education, and source of information and economic factors like household income could affect dietary diversity significantly. As the study was conducted by interviewing mothers on the past 24-hour feeding practice for their 6–23-month-old children, there might be a recall bias and the study does not address the amount and quality of the complementary food.

## 5. Conclusion

This study has shown that approximately half of the mothers have good knowledge on minimum dietary diversity for 6–23-month-old children and that very low proportion of 6–23-month-old children receive diversified meal according to Infant and Young Child Feeding indicators. In general, minimum dietary diversity practice for 6–23-month-old children was below the Infant and Young Child Feeding recommendation. It was identified that mothers' knowledge was affected by mothers' age, husbands' education status, marital status, household income, and source of health information. Mothers' dietary diversity practice was also found to be affected by different sociodemographic and economic factors such as mother's age, marital status, and seasonal availability of all food groups and strongly associated with the source of health information. The association found between knowledge and practice outcome suggests that increasing knowledge of mothers on dietary diversity could improve dietary diversity practice of mothers for 6–23-month-old children. Based on the finding of this study, the following recommendations were forwarded to different stakeholders:

- Health sector needs to design a community-based nutrition focused behavioral change communication at a community level to equip and enhance community knowledge and to change knowledge into practice of proper dietary diversity for 6–23-month-old children.
- Enhancing health workers' knowledge and skill in transferring nutrition-based health information for mothers at health center level could help mothers who receive health information from HC to practice dietary diversity properly.
- Media has to strengthen social behavioral change and communication at community level to change knowledge on dietary diversity to practice. In addition, animal source food utilization has to be promoted.
- Government has to urge partners working in the area of nutrition to promote mobile kitchen to promote food cooking demonstration at community level which could strengthen communities' skill in practicing dietary diversity using locally available foods.
- Ministry of Women and woreda women offices needs to promote mothers education to enhance mothers' knowledge and increase level of dietary diversity practice.
- In order to make all food groups at household level available throughout all the seasons, Agriculture Bureau and Office needs to promote home stead gardening and permagarden to produce vegetables and fruits at the HH level. This will improve access to fruits and vegetables throughout the year and improve DD of children.
- Ministry of Education and Education Office have to expand education to all levels and strengthen education for all especially teaching husbands which plays a significant role in changing wives'/mothers' knowledge level.
- Further study has to be conducted in the area to determine adequacy of nutrient intake and on comparison of nutrient intake and nutritional status of 6–23-month-old children.

## Figures and Tables

**Table 1:** Sociodemographic and socioeconomic characteristics of respondents, Adea woreda, East Shoa Zone, Oromia region, 2014.

Variables	Number	Percent (%)
Mother's age		
15–24 years	176	25.1
35–44 years	437	62.4
25–34 years	87	12.4
Child's age in months		
6 to 11 months	203	29.0
12 to 18 months	409	58.4
19–23 months	88	12.6
Mother's educational status		
No schooling	220	31.4
Primary	424	60.6
Secondary and above	56	8
Husband's educational status		
No schooling	95	13.6
Primary	468	66.9
Secondary and above	137	19.5
Religion		
Orthodox	489	69.9
Muslim	72	10.2
Protestant	139	19.9
Marital status		
Single	19	2.7
Married	658	94.0
Divorced	23	3.3
Family size		
Below five	242	34.6
Five and above	458	65.4
Number of 6–23-month-old children in the HH		
One	666	96.0
Two and above	34	4.9
Monthly income		
501 to 1000 birr	391	55.9
1001 to 3000 birr	309	44.1
HH economy controlled by		
Husband only	645	92.1
Housewife only	30	4.3
Both husband and wife	25	3.6

HH: household.

**Table 2:** Knowledge level of the interviewed mothers on minimum dietary diversity in Adea woreda, East Shoa Zone, Oromia region, November 2014.

Knowledge responses	Number	Percent %
Age when complementary feeding was initiated		
At the sixth month	670	95.7
Other	30	4.3
Importance of giving additional food		
Breast milk alone is not sufficient	589	84.1
Other	111	15.9
Pictures of porridge the mother chose		
Show thick porridge	61	8.7
Show watery porridge	639	91.3
Type of food added to porridge		
Three and above groups	253	36.1
Less than three groups	447	63.9
Recommendation on bottle feeding with nipple		
No	127	18.1
Yes	573	81.9
Knowledge status of mother		
Not knowledgeable	343	49
Knowledgeable	357	51

**Table 3:** Level of practice of the interviewed mothers on minimum dietary diversity for 6–23-month-old children, Adea woreda, East Shoa Zone, Oromia region, 2014.

Practice assessing questions responses	Number	Percent %
Child drank juice or juice drinks yesterday during day or night		
No	686	98
Yes	14	2
Child ate injera, bread, rice made from teff, oat, maize, barley, wheat		
No	37	5.3
Yes	663	94.7
Child ate tinned, powdered, fresh animal milk or yoghurt yesterday		
No	393	56.1
Yes	307	43.9
Child ate pumpkin, carrot, squash, or sweet potato that are yellow		
No	677	96.7
Yes	23	3.3
Child ate ripe mango or papaya yesterday during day or night		
No	686	98
Yes	14	2
Child ate any dark green leafy vegetables like kale, spinach		
No	541	77.3
Yes	159	22.7
Child ate any other fruit or vegetables yesterday during day or night		
No	558	79.7
Yes	142	20.3
Child ate meat of ox, pig, hen, sheep, or calf yesterday during day or night		
No	632	90.3
Yes	68	9.7
Child ate rgg yesterday during day or night		
No	358	51.1
Yes	342	48.9
Child ate any foods made from beans, peas, lentils, or nuts yesterday		
No	126	18
Yes	574	82
Dietary diversity practice status of mother		
Not good	588	84
Good	112	16

**Table 4:** Factors associated with knowledge of mothers towards dietary diversity in Adea district, Ethiopia, 2014.

Variable	Knowledge status		Crude odds ratio (95% CI)	Adjusted OR (95% CI)	*P* value adjusted
	Knowledgeable	Not knowledgeable			
Mother's age in year					
15–24 years	85	91	1		
25–34 years	243	194	1.34 (0.94, 1.90)	1.27 (0.86, 1.87)	0.233
35–44 years	29	58	0.54 (0.31, 0.91)	0.46 (0.26, 0.83)^*∗*^	0.010
Husband's education status					
Cannot read and write	38	57	1		
Primary	226	242	1.40 (0.89, 2.19)	1.23 (0.76, 1.98)	0.404
Secondary and above	93	44	3.17 (1.84, 5.47)	2.79 (1.55, 5.00)^*∗*^	0.001
Marital status					
Single	15	4	1		
Married	328	330	0.27 (0.09, 0.81)	0.17 (0.05, 0.56)^*∗*^	0.002
Divorced	14	9	0.42 (0.10, 1.66)	0.35 (0.08, 1.52)	0.161
Season in which all food is available					
Summer	77	97	1		
Winter	280	246	1.43 (1.02, 2.02)	1.46 (1.01, 2.12)^*∗*^	0.047
Household income					
501–1000 birr	170	221	1		
1001–3000 birr	187	122	1.99 (1.47, 2.70)	1.79 (1.28, 2.51)^*∗*^	0.001
Source of health information					
From health post	85	101	1		
From Health center	15	40	0.47 (0.23, 0.86)	0.47 (0.23, 0.93)^*∗*^	0.030
HP & health development army	167	161	1.23 (0.59, 1.77)	1.14 (0.78, 1.67)	0.504
HP and media	90	41	2.61 (1.63, 4.17)	2.25 (1.37, 3.69)^*∗*^	0.001

*Note.* ^*∗*^Statistically significant at *P* value < 0.05 and 95% CI. 1: reference category.

**Table 5:** Factors associated with mothers' dietary diversity practice in Adea district, Ethiopia, 2014.

Variable	DD practice		Crude odds ratio (95% CI)	Adjusted OR (95% CI)	*P* value adjusted
	Good	Not good			
Mother's age in year					
15–24 years	21	155	1		
25–34 years	81	356	1.68 (1.00, 2.81)	1.82 (1.05, 3.17)^*∗*^	0.026
35–44 years	10	77	0.96 (0.43, 2.14)	1.29 (0.55, 3.03)	0.553
Marital status					
Single	9	10	1		
Married	101	557	0.20 (0.80, 0.51)	0.22 (0.08, 0.59)^*∗*^	0.003
Divorced	2	21	0.11 (0.02, 0.58)	0.09 (0.02, 0.53)^*∗*^	0.008
Season in which all food is available					
Summer	19	155	1		
Winter	93	433	1.75 (1.04, 2.97)	1.78 (1.01, 3.15)^*∗*^	0.046
Source of health information					
From health post	41	145	1		
From health center	4	51	0.28 (0.10, 0.81)	0.36 (0.12, 1.10)	0.073
HP & health development army	42	286	0.52 (0.32, 0.84)	0.48 (0.29, 0.79)^*∗*^	0.004
HP and media	25	106	0.83 (0.48, 1.46)	0.68 (0.37, 1.22)	0.192
Knowledge status					
Knowledgeable	85	272	1		
Not knowledgeable	27	316	0.27 (0.17, 0.43)	0.30 (0.18, 0.48)^*∗*^	0.000

*Note.* ^*∗*^Statistically significant at *P* value < 0.05 and 95% CI. 1: reference category.
